# Quality Evaluation of Traditional Chinese Medicine Compounds in Xiaoyan Lidan Tablets: Fingerprint and Quantitative Analysis Using UPLC-MS

**DOI:** 10.3390/molecules21020083

**Published:** 2016-01-22

**Authors:** Na Yang, Aizhen Xiong, Rui Wang, Li Yang, Zhengtao Wang

**Affiliations:** 1 The MOE Key Laboratory for Standardization of Chinese Medicines and The SATCM Key Laboratory for New Resources and Quality Evaluation of Chinese Medicines, Institute of Chinese Materia Medica, Shanghai University of Traditional Chinese Medicine, Shanghai 201203, China; yn__1988@126.com (N.Y.); a.z.xiong@hotmail.com (A.X.); ztwang@shutcm.edu.cn (Z.W.); 2 Shanghai R & D Centre for Standardization of Chinese Medicines, Shanghai 201203, China; 3 School of Pharmacy, Shanghai University of Traditional Chinese Medicine, Shanghai 201203, China

**Keywords:** chromatographic fingerprint, XiaoyanLidan tablets, formulae, UPLC-MS, traditional Chinese medicine

## Abstract

XiaoyanLidan tablets (XYLDTs) are traditional Chinese medicines frequently used for syndromes of the liver and gallbladder, cholecystitis and cholangitis. To evaluate the consistency of the quality of commercial XYLDT preparations, we established a simple and reliable ultra-performance liquid chromatography (UPLC) method with a photodiode array (PDA) detector and mass spectrometry (MS), including a fingerprint analysis and quantification of the main pharmacologically-active markers. In the UPLC-PDA detection-based fingerprint analysis of XYLDTs, approximately 39 peaks were found in the XYLDT chromatogram, 26 of which were attributed to *Picrasmaquassioides*, nine to *Andrographis* and four to *Isodonserra*. Subsequently, the structures of these bioactive markers were identified through ESI-MS analyses. Using the chemometricmethods of similarity analysis and principal component analysis, the five significant herbal componentswere determined as 4-methoxy-5-hydroxycanthin-6-one, andrographolide, dehydroandrographolide, neoandrographolide and rosmarinic acid, and these components were qualitatively assessed. Our experimental results demonstrated that combining the fingerprint analysis with UPLC-MS and multi-ingredient determination is useful for rapid pharmaceutical quality evaluation. Moreover, the combined approach can potentially differentiate the origin, determine the authenticity and assess the overall quality of the formulae.

## 1. Introduction

Because diagnosis and treatment in traditional Chinese medicine (TCM) are based on the concepts of Yin-Yang, the five-element theory and long-term clinical experience, TCM-based healthcare has been highly recommended. TCM is characterized by its unique theoretical system and entails complex components that are effective through their synergistic effects. Currently, the quality of the preparation and feeding herbs cannot be evaluated sufficiently and systematically. Therefore, we should establish a systematic and comprehensive system to improve the preparation and evaluate the quality of TCM. According to the pharmacopeia, XiaoyanLidan tablets (XYLDTs) comprise three types of TCM: *Andrographis*, *Picrasma quassioides* and *Isodonserra*. These XYLDTs are mainly used to clear away heat and to expel dampness from the body in traditional Chinese medicine and function as cholagogues. They play crucial roles in treating bitter taste, hypochondriac pain caused by wetness-heat of the liver, gallbladder syndrome, acute cholecystitis and cholangitis. Andrographolide, neoandrographolide and rographolide are standards of *Andrographis* for qualitative evaluation, and andrographolide and neoandrographolide are standards of *P. quassioides* and *Isodonserra* for quantitative evaluation. Only reference crude herbs and no specific standards for qualitative and quantitative evaluation exist [1].

The chemical composition of XYLDTs is complex. *Andrographis* mainly contains lactones and flavonoids. The main components of *P. quassioides* include alkaloids, bitter principles, triterpenoids and volatile oil. *Isodonserra* mainly contains flavonoids, terpenoids, sterols, microelements and volatile oil. Andrographolide, neoandrographolide and dehydroandrographolide have recently been shown to be the biological components of *Andrographis*, which exhibit anti-inflammatory, antiviral, antitumor, antibacterial, hepatoprotective, analgesic, antiradical and anti-HIV effects [2,3,4,5,6,7,8,9,10,11,12,13]. In addition, 4-methoxy-5-hydroxycanthin-6-one and rosmarinic acid are crucial in the medicinal effects of XYLDTs. 4-methoxy-5-hydroxycanthin-6-one has substantial antibacterial properties [14], exhibits high inhibitory activity against cyclic adenosine monophosphate phosphodiesterase [15] and increases the intestinal blood flow rate [16]. It also inhibits ulcerative colitis induced by dextran sulfate sodium in rats [17]. A previous study evaluated the anti-inflammatory effect of 4-methoxy-5-hydroxycanthin-6-one, including the *in vitro* inhibitory effect on the production of nitric oxide, the release of tumor necrosis factor-alpha in lipopolysaccharide-activated macrophages and an *in vivo* inhibitory effect on the development of xylene-induced ear edema in mice, carrageenan-induced paw edema in rats, as well as adjuvant-induced arthritis in rats [18]. Rosmarinic acid has several neuroprotective properties, including an antidepressant-like effect [19].

Quality control is the most critical issue related to clinical medication safety. The theory of the therapeutic effect of TCM states that the effect of a compound is based on its synergistic effect and the antagonism of its multiple constituents [20,21,22,23]. Because substantial differences may exist in the quality and quantity of chemical components, detecting only one or two “effective” compounds is insufficient for effective quality control. Now, fingerprinting is comprehensively used to analyze food, herbs, as well as their preparations in various fields world widely [24,25,26]. Chromatographic fingerprint technology is currently used to comprehensively and qualitatively analyze the complex components of TCM. Additionally, it is an effective method for evaluating the merits, identifying the authenticity, differentiating species and ensuring the consistency and stability of TCM. HPLC is used to establish the chromatographic fingerprint. However, the process is time consuming because of the complex contents of the formulae. By contrast, ultra-performance LC-MS (UPLC-MS) is characterized by desirable parameters, such as short analysis time, high sensitivity and precision and quantitative accuracy.

Yao *et al.* [27] established an HPLC fingerprint chromatogram for XYLDTs for evaluating their quality. Some peaks were identified by comparing them tothe retention time (RT) of the chemical reference substance. Moreover, evaluating the quality of 24 batches of samples from three different manufacturers through a TCM fingerprint similarity evaluation system revealed several similarities between the commercial products because of dehydroandrographolide. Jin *et al.* [28] developed a rapid HPLC-MS method for simultaneously quantitatively determining six chemical XYLDT components, namely andrographolide, dehydroandrographolide, lasiodonin, epinodosinol, oridonin and epinodosin, in 15 min. However, these studies have some disadvantages. The sample size was too small to determine statistical differences among different manufacturers. Moreover, the index components for quantitation were not the key medicinal components. Therefore, studies, including a sufficient sample size and preparations representative of the wide range available on the market and using simple methods with a short analysis time and representative index components for quantitation, are urgently required.

In this study, using UPLC-MS, we developed an analysis method for specific, practical and reliable chromatographic fingerprinting of XYLDTs. This study investigated the relationship among XYLDTs from different manufacturers, clarified most of the chemical components of the preparation and attributed 39 peaks to three herbs constituting the preparation by comparing the chromatograms of three overlapping TCM extracts. Most structures of the main herb markers were identified through targeted UPLC-ESI-MS analyses. Meanwhile, similarity analysis (SA) and principal componentanalysis (PCA) were performed to classify and discriminate the samples from different manufacturers and to identify the chemical markers. We quantitatively assessed the concentrations of five components. To the best of our knowledge, this is the first study to compare the differences in samples from different manufacturers and to report the constituents relative to the three original herbs by using non-targeted UPLC fingerprinting following targeted UPLC-MS analysis.

## 2. ExperimentalSection

### 2.1. Plant Materials and Reagents

**Figure 1:**
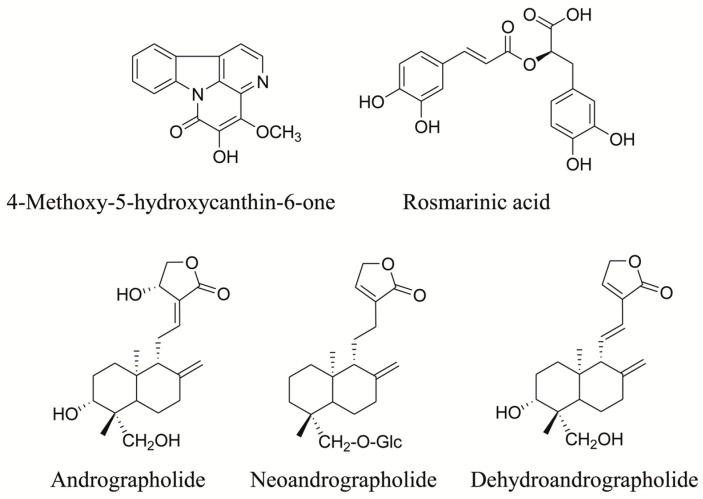
Chemical structures of the five bioactive components of XYLDTs.

A total of 113 samples of XYLDTs were obtained from different manufacturers, including LFS, BYS, WNQ, JY, XF, JK, BH, GF, JM, YH, QZ, LS and QT. The reference compounds are shown in Figure 1. Andrographolide, dehydroandrographolide, neoandrographolide and rosmarinic acid were obtained from Shanghai R & D Center for Standardization of Chinese Medicines (Shanghai, China); 4-methoxy-5-hydroxycanthin-6-one was isolated from the stem of *P. quassioides*, and the structure was elucidated using MS, ^1^H- and ^13^C-NMR spectroscopy and confirmed by comparing the data with those in the literature. The purity of all compounds, determined through HPLC-UV analyses, exceeded 95%. HPLC-grade acetonitrile and formic acid (FA) (Merck, Darmstadt, Germany) were used for the preparation of the mobile phase. Purified water was prepared using a Mini-Q water-purification system (Millipore, Bedford, MA, USA). Other solvents used for analyses were of analytical grade.

### 2.2. Apparatus and Analytical Methods

UPLC analyses were performed using a Waters ACQUITY UPLC™ system (Waters Corporation, Milford, CT, USA) equipped with a binary solvent delivery system, an autosampling manager, a column compartment, a photodiode-array detection (PDA) system and a TOD™ Mass spectrometer (Waters Corporation) equipped with an electrospray interface (ESI).

The samples were separated on a Waters Acquity BEH C_18_ column (100 mm × 2.1 mm i.d., 1.7 μm) at a temperature of 30 °C. Mobile Phase A was acetonitrile, while Mobile Phase B was 0.1% FA solution. A gradient program was chosen as follows: 0–2 min, linear gradient 10%–15% A; 2–3 min, linear gradient 15%–18% A; 3–10 min, linear gradient 18%–25% A; 10–14 min, linear gradient 25%–27% A; 14–25 min, linear gradient 27%–50% A; 25–30 min, isocratic 50% A. The flow rate was 0.3 mL/min; the absorbance was monitored at 254 nm, and the volume of injected sample and standard solutions was 5 μL.

The MS spectra was operated in positive ion mode with the scan, working under the following conditions: the full-scan mass spectra were recorded from *m/z* 100 to *m/z* 1000; capillary voltage: 3.0 V; cone voltage: 40 kV; extractor voltage: 3.0 kV; source temperature: 120 °C, desolvation temperature: 400 °C; desolvation gas (nitrogen): 700 L/h; cone gas (nitrogen): 50 L/h.

### 2.3. Sample Preparation

The pretreatment of the sample was that 10 tablets of sample were removed from the coating and then pulverized for use. For the extraction, 0.3 g of the dried powder wereadded into 50 mL of methanol, which contained 0.5% hydrochloric acid at 30 °C in an ultrasonic water bath for 30 min and then was cooled at room temperature. The solution was filtered through a 0.22-μm membrane filter, and the filtered solution was used as the sample solution.

### 2.4. Data Analyses

The UPLC-UV (254 nm) chromatographic data in the Waters instrument-specific format (.dat) were exported as AIA files (*.cdf) and analyzed using professional software (Similarity Evaluation System for Chromatographic Fingerprint of Traditional Chinese Medicine, China Committee of Pharmacopeia, 2.0).

The area of each peak is expressed as a percentage against the total peak area, that is the percentage peak area (PPA). The PPA of each peak was calculated and used for differentiating the samples. Chromatographic fingerprints developed for the 113 samples obtained from 13 different manufacturers were processed as described, and the PPAs of the peaks were statistically analyzed using SPSS 18.0 and Soft Independent Modeling of Class Analogy-P 13.0.

## 3. Results and Discussion

### 3.1. Optimization of Extraction and UPLC Conditions

The conditions for sample pretreatment were optimized by investigating the type of solvent (methanol or ethanol), the solvent concentration in water (70%, *v*/*v*), the hydrochloric acid concentration in methanol (0.5%, *v*/*v*) and extraction time (10, 20, 30 and 60 min). Considering the types and quality of the samples, hydrochloric acid concentration in methanol (0.5%, *v*/*v*) was the ideal extraction solvent that effectively extracted all compounds (Figure S1). A study of the influence of the extraction time on the extraction efficiency showed that most marker compounds were extracted with the highest yield within 30 min. Ultrasonication times longer than 30 min did not substantially increase the quantity of the components in the extracts.

Several chromatographic parameters, including the chromatographic column, mobile phase composition, wavelength and column temperature, were optimized for the UPLC analysis to achieve a high resolution in as short a time as possible. After testing many columns, including ACQUITY UPLC HSS C_18_ column (100 × 2.1 mm, i.d., 1.7 µm), ACQUITY UPLC BEH C_18_ column (100 × 2.1 mm, i.d., 1.7 µm) and Kinetex 1.7 µm XB-C_18_ 100A (100 × 2.1 mm, i.d., 1.7 µm), finally, the ACQUITY UPLC BEH C_18_ column (100 × 2.1 mm, i.d., 1.7 µm) was used in the analysis (Figure S2). Compared to methanol–water, acetonitrile–water yielded a high resolution. The addition of FA at 0.1% (*v*/*v*) achieved a satisfactory baseline peak shape and resolution. Satisfactory separation was achieved in 30 min through gradient elution under UPLC conditions, as described in Section 2.2. Considering the number of peaks, quantitation and sensitivity, the detector wavelength was set at 254 nm.

### 3.2. UPLC Fingerprinting of XYLDT Samples and Crude Herbs

**Figure 2:**
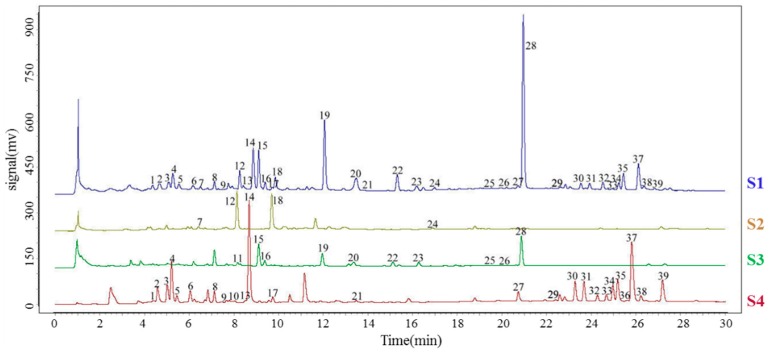
UPLC-UV fingerprint chromatograms of XYLDTs (S1) and three crude herbs, Isodon serra (S2), Andrographis (S3), and Picrasma quassioides (S4).

The optimized UPLC method with a photodiode array (PDA) detector was used for enhanced separation, identificationand recovery of the chromatograms of the studied samples according to the aforementioned method. In total, 113 batches of XYLDT samples and three types of crude herbs were analyzed. Additionally, their chromatograms were introduced in the aforementioned professional software. The reference chromatographic fingerprints of a typical UPLC chromatogram of the XYLDT samples and crude herbs are presented in Figure 2. The fingerprints revealed the presence of approximately 39 common peaks in the chromatogram of XYLDTs. All peaks were identified as components of the three crude herbs. All samples generally presented consistent chromatographic patterns, but varied in peak abundances. Peak 14 (RT = 8.7 min), a component with a consistently high concentration, was found in 113 chromatograms. Therefore, this peak was used as the reference peak for calculating the relative retention time (RRT) and relative peak area (RPA) for the other characteristic peaks. The average RRT and RA values for XYLDTs were calculated.

### 3.3. Identification of the Structures of the Main Herb Markers Using ESI-MS

To characterize the main peaks of the fingerprint chromatograms, ESI-MS was combined with UPLC analysis; 15 peaks were identified on the basis of their RT, PDA spectra and MS spectra. Among the peaks, Peaks 14, 15, 22, 24 and 28 were identified as the five components, including 4-methoxy-5-hydroxycanthin-6-one, andrographolide, neoandrographolide, rosmarinic acid and dehydroandrographolide, which were compared toreference standards and the related literature, shown as Table 1 [28,29,30,31], and the others were compared to therelated literature, shown as Table 1 [28,29,30,31].

**Table 1:** Characterization of 15 compounds identified from XYLDT.

Peak Number	RT	MS (*m/z*) [M + H]^+^	MS2 (*m/z*) [M + H]^+^	Compounds
***Picrasmaquassioides***				
2	5.43	251	168	3-Methylcanthin-5,6-dione
5	6.45	243	213	1-ethyl-4-Methoxy-8-hydroxycanthin-β-carboline
9	8.66	257	197	4,8-Dimethoxyl-1-ethyl-carboline
14	9.67	267	168	4-Methoxy-5-hydroxycanthin-6-one
23	16.89	281	237	4,5-Dimethoxycanthin-6-one
27	21.74	241	213	1-Ethenyl-4-methoxy-8-hydroxycanthin-β-carboline
29	22.51	481	253	Picradidine H
30	24.04	495	253	Picradidine C
31	24.85	225	197	1-Ethenyl-4-methoxy-β-carboline
33	25.81	479	267	Picradidine F
37	26.86	255	197	1-Ethenyl-4,8-dimethoxyl-β-carboline
***Andrographis***				
15	10.16	351	211	Andrographolide
22	17.55	481	237	Neoandrographolide
28	22.13	333	297	Dehydroandrographolide
***Isodonserra***				
24	17.57	361	139	Rosmarinic acid
others	-	-	-	Unknown

### 3.4. SA and PCA

The reference chromatographic fingerprint was generated on the basis of 113 XYLDT samples containing three herbs obtained from 18 different manufacturers. Additionally, their representative chromatograms are illustrated in Figure S3. Using the reference chromatographic fingerprint, we conclude that the principle constituents of XYLDTs are relatively homogeneous. Subsequently, intra-manufacturer similarity comparison was performed. Using the professional software, UPLC fingerprints of samples from different manufacturers were correlated with their corresponding digital standard fingerprint (R), and the correlation coefficients were calculated. The results (Figure S4 and Table S1) show that all samples from the same manufacturer were highly correlated with the reference chromatographic fingerprint. Therefore, we concluded that the quality of the samples from the same manufacturer is consistent, and the craftsmanship of the manufacturer is stable. PCA, an unsupervised multivariate data analysis approach, is appropriate when the function of many attributes is involved in different samples. PCA was performed on the PPA to ensure that all elements equally influenced the results [29]. Through the eigenvalues and percentage variance determined for each PC (Figure S5 and Table S2), we conclude that the first seven PCs explain 87.2% of the relevant information.

To analyze the relationship among all samples, we performed a data array of the fingerprints, which is a 113 × 39 data matrix including all samples. In this matrix, each crosswise represented all peaks of a sample, and the columns represented the areas of the same detected peaks for each sample. A score plot was derived from the PCA, in which each marker represented a sample; highly consistent samples were clustered into 13 groups.

**Figure 3:**
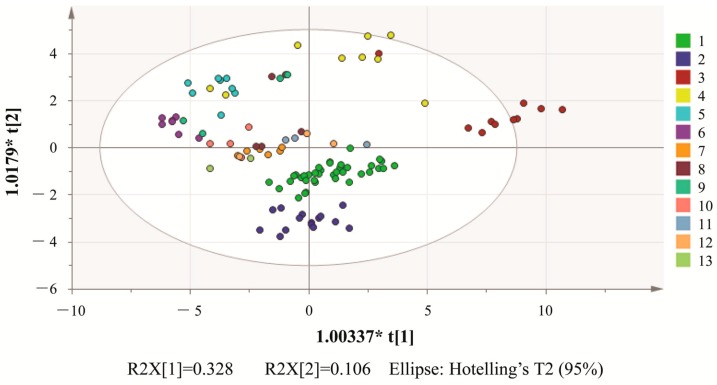
The scores plot obtained by PCA of the 113 samples, LFS (1), BYS (2), WNQ (3), JY (4), XF (5), JK (6), BH (7), GF (8), JM (9), YH (10), QZ (11), LS (12) and QT (13).

**Figure 4:**
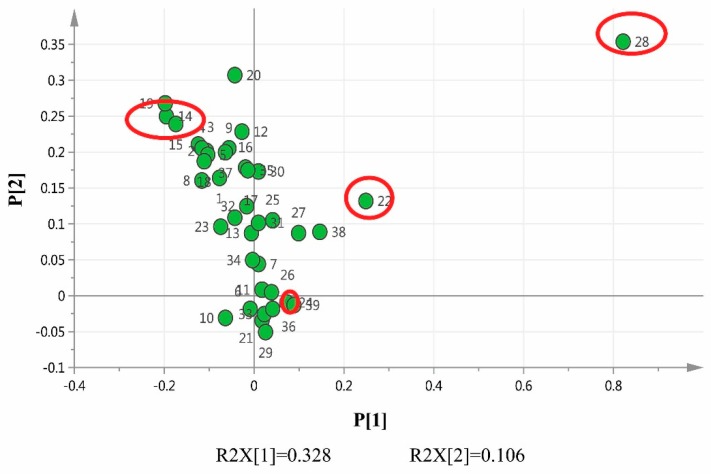
The loading plot for the common peaks, 4-methoxy-5-hydroxycanthin-6-one (14), andrographolide (15), neoandrographolide (22), rosmarinic acid (24) and dehydroandrographolide (28).

From the unsupervised PCA classifications (Figure 3), we can only classify and discriminate the samples on the basis of the concentrations of the components. However, we cannot accurately identify which components influenced the discrimination more than the others. The loading plot of PCA (Figure 4) indicated that five peaks, namely 14, 15, 22, 24 and 28, played crucial roles in differentiating the samples. From the results of the aforementioned identification, the five components are 4-methoxy-5-hydroxycanthin-6-one, andrographolide, neoandrographolide, rosmarinic acid and dehydroandrographolide, which can be used as markers to determine the internal quality of the samples and their origins.

### 3.5. Analytical Characterizationof the Quantitative Analysis

The concentrations of the five components, including the four marker components, namely 4-methoxy-5-hydroxycanthin-6-one, rosmarinic acid, andrographolide, dehydroandrographolide and neoandrographolide, were quantitatively determined through UPLC-PDA. The developed UPLC-PDA method was validated by assessing the precision, repeatability, stabilityand recovery. The calibration curves reflect a good linearity with a high correlation coefficient within the tested concentration ranges; the results are shown in Table 2, Table 3 and Table 4.

The calibration curves of the five components for quantitation were created by establishing a relationship between the peak area (Y) and the concentration (X, μg/mL) of the standard solution. The data of all calibration curves illustrated a satisfactory linearity (*r*^2^ > 0.9999) within the tested concentration ranges. The precision tests were performed using six replicated injections of the same sample solution (No. 53) in a day. Variations were less than 3.00% (range: 0.11%–2.94%). The repeatabilityof the method was determined by six independently-prepared sample solutions (No. 53). Variations were less than 3.00% (range: 0.54%–2.78%). The stability of the method was examined in triplicate for threeconsecutive days with one sample solution (No. 53). Variations were less than 3.00% (range: 0.18%–2.61%). Recoveries were operated as follows: the addition of the five standard solutions (quantities of which were accurately known from the previously-analyzed sample solution), followed by the reanalysis of the sample solution. Each analysis was repeated nine times. The results revealed a high accuracy, with overall recoveries exceeding 95% (range: 97.2%–103.3%).

**Table 2:** Precision, reproducibility and stability for the analysis of the five bioactive compounds.

Compound	Precision (*n* = 6)		Reproducibility (*n* = 6)		Stability (*n* = 6)	
	Content (mg·Tab^−1^)	RSD (%)	Content (mg·Tab^−1^)	RSD (%)	Content (mg·Tab^−1^)	RSD (%)
4-Methoxy-5-hydroxycanthin-6-one	0.5273	2.94	0.5394	2.78	0.5414	2.61
Andrographolide	8.4279	0.11	8.3933	0.54	8.4387	0.18
Dehydroandrographolide	4.4402	0.20	4.5892	1.55	4.5711	0.80
Neoandrographolide	2.5032	2.14	2.5326	1.68	2.4955	1.70
Rosmarinic acid	2.2165	0.30	2.2200	0.66	2.2264	0.62

**Table 3:** Recovery for the analysis of the five bioactive compounds.

Compound	Original (mg)	Spiked (mg)	Found (mg)	Recovery (%)	Average Recovery (%)	RSD (%) (*n*= 3)
4-Methoxy-5-hydroxycanthin-6-one	0.3236	0.1511	0.4827	103.7	102.4	2.54
		0.3022	0.6304	100.0		
		0.4533	0.7966	103.4		
Andrographolide	5.0360	2.5444	7.6376	100.8	100.1	1.32
		5.0888	10.2118	100.3		
		7.6332	12.6637	99.1		
Dehydroandrographolide	2.7535	1.3812	4.1851	102.2	103.3	1.53
		2.7624	5.6323	102.8		
		4.1436	7.1293	104.8		
Neoandrographolide	1.5196	0.7509	2.2602	97.2	97.2	1.02
		1.5018	3.0400	99.8		
		2.2527	3.8956	104.6		
Rosmarinic acid	1.3320	0.6505	1.9989	101.0	100.9	2.44
		1.3010	2.6874	98.0		
		1.9515	3.2809	102.1		

**Table 4:** Calibration curves for the analysis of the five bioactive compounds.

Compound	Regression Equation (*Y* = a*x* + b)	*r*^2^	Linear Range (μg·mL^−1^)
4-Methoxy-5-hydroxycanthin-6-one	*Y* = 97672*x* − 5.023	0.9999	0.50–32.16
Andrographolide	*Y* = 31564*x* − 6.875	0.9999	10.00–160.02
Dehydroandrographolide	*Y* = 37545*x* − 10.8	1.0000	40.13–200.65
Neoandrographolide	*Y* = 27057*x* + 19.806	1.0000	17.03–122.64
Rosmarinic acid	*Y* = 26300*x* + 1.9303	0.9999	1.91–61.25

### 3.6. Quantitation of 4-Methoxy-5-hydroxycanthin-6-one, RosmarinicAcid, Andrographolide, Dehydroandrographolide and Neoandrographolide

The developed UPLC method was successfully applied to quantify the five components of the XYLDT samples collected from different manufacturers. The findings showed that the concentrations of the five compounds differed. The concentrations ranges are as follows: 4-methoxy-5-hydroxycanthin-6-one, 0.01–2.41 mg/g; rosmarinic acid, 0.57–10.14 mg/g; andrographolide, 2.35–34.96 mg/g; dehydroandrographolide, 10.81–56.68 mg/g; and neoandrographolide, 4.84–32.79 mg/g.

**Figure 5:**
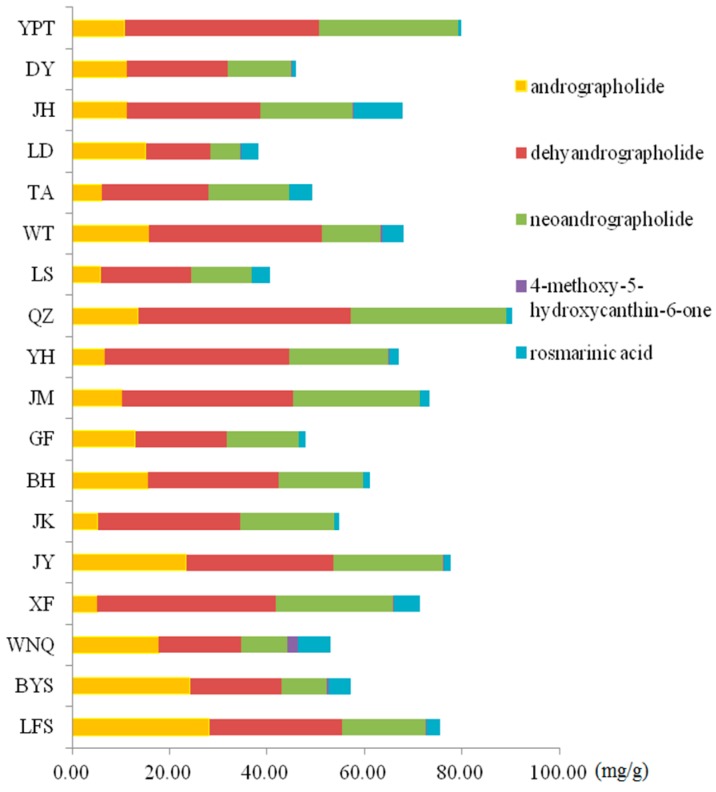
The bar graph of the average concentrations of five components in samples from 18 manufactures.

Figure 5 shows the average concentration of the same compound for different batches of samples from the same manufacturer. This average concentration showed quality diversity, possibly because of the different origins of the three types of crude medicine. The samples from WNQ contain high levels of 4-methoxy-5-hydroxycanthin-6-one compared tothe other samples, and the samples from LFS contain high levels of andrographolide compared tothe other samples. The proportions of andrographolide, dehydroandrographolide and neoandrographolide are different, possibly because of the different origins of the crude medicine and the production technology of XYLDTs. According to previous studies, andrographolide, dehydroandrographolide and neoandrographolide exhibit different pharmacological effects and toxic effects [32], and the pharmacopeia only indicates quality control for rographolide and quantity evaluation for andrographolide and dehydroandrographolide. Therefore, detecting the proportion of the three compounds simultaneously is necessary.

## 4. Conclusions

Compared toYao *et al.* [27], our sample size was more sufficient and representative ofthe market with simpler methods and shorter analysis time. We are clearly aware of the differences of different manufactures and the differences between different batches of the same manufacture. Compared to Jin *et al*. [28], the index components for quantitation were key medicinal components and more pertinent.

In summary, we first established the UPLC fingerprint analysis to determine most constituents of XYLDTs and to compare the difference in the samples from different manufacturers. Meanwhile, by comparing the MS characteristics obtained through UPLC-PDA-MS and the existing literature, 15 components were identified. The PCA results clustered the samples into groups, implying that the sample sources are highly consistent. Moreover, the SA results revealed that the samples from the same manufacturer had high relativities. From the PCA loading plot, the greatest impact factors of the constituents characteristic to the classification were identified. Furthermore, the concentrations of the five main components, namely 4-methoxy-5-hydroxycanthin-6-one, rosmarinicacid, andrographolide, dehydroandrographolide and neoandrographolide, were quantitatively determined. The analytical methods were validated as rapid, sensitive, precise and accurate. The concentrations of the five components differed, with considerable differences in the concentrations of andrographolide, dehydroandrographolide and neoandrographolide. This finding suggests that both the origins of the crude herbs and the production technology should be controlled.

## Supplementary Materials

Supplementary materials can be accessed at: http://www.mdpi.com/1420-3049/21/1/83/s1.

## Abbreviations:

The following abbreviations are used in this manuscript:

XYLDTs: XiaoyanLidan tablets
UPLC: ultra-performance liquid chromatography
PDA: photodiode array
SA: similarity analysis
PCA: principal component analysis
