# Supplementary material for: Quality Evaluation of Traditional Chinese Medicine Compounds in Xiaoyan Lidan Tablets: Fingerprint and Quantitative Analysis Using UPLC-MS
Source: Molecules. 2016 Jan 22;21(2):83. doi: 10.3390/molecules21020083 (PMC6273587; doi:10.3390/molecules21020083)
Supplement: Supplementary file 1 [file molecules-21-00083-s001.pdf]

Supplementary Materials: Quality Evaluation of Traditional Chinese Medicine Compounds in Xiaoyan Lidan Tablets: Fingerprint and Quantitative Analysis Using UPLC-MS

Na Yang [1], Aizhen Xiong [1,2], Rui Wang [2,3,*], Li Yang [1,2,*] and Zhengtao Wang [1,2]

Table S1. (**A**) The similarity of Xiaoyan Lidan tablets from LFS.

Sample No.	Similarity	Sample No.	Similarity
L01	0.996	L20	0.997
L02	0.999	L21	0.974
L03	0.956	L22	0.997
L04	0.999	L23	0.999
L05	0.994	L24	0.995
L06	0.994	L25	0.997
L07	0.998	L26	0.992
L08	0.999	L27	0.997
L09	0.997	L28	0.997
L10	0.999	L29	0.999
L11	0.994	L30	0.998
L12	0.994	L31	0.995
L13	0.982	L32	0.961
L14	0.968	L33	0.985
L15	0.999	L34	0.973
L16	0.999	L35	0.991
L17	0.995	L36	0.995
L18	0.999	L37	0.996
L19	0.993		

Table S1. (**B**) The similarity of Xiaoyan Lidan tablets from BYS.

Sample No.	Similarity	Sample No.	Similarity
L38	0.990	L46	0.991
L39	0.997	L47	0.998
L40	0.995	L48	0.990
L41	0.994	L49	0.991
L42	0.997	L50	0.984
L43	0.989	L51	0.996
L44	0.982	L52	0.994
L45	0.998		

Table S1. (**C**) The similarity of Xiaoyan Lidan tablets from WNQ.

Sample No.	Similarity	Sample No.	Similarity
L63	0.991	L68	0.999
L64	0.985	L69	0.998
L65	0.997	L70	0.998
L66	0.999	L71	0.995
L67	0.996		

Table S1. (**D**) The similarity of Xiaoyan Lidan tablets from JY.

Sample No.	Similarity	Sample No.	Similarity
L72	0.999	L76	0.996
L73	0.999	L77	0.999
L74	0.999	L78	0.888
L75	0.999	L79	0.984

Table S1. (**E**) The similarity of Xiaoyan Lidan tablets from XF.

Sample No.	Similarity	Sample No.	Similarity
L53	0.999	L58	1
L54	0.999	L59	1
L55	0.998	L60	1
L56	1	L61	1
L57	1	L62	0.999

Table S1. (**F**) The similarity of Xiaoyan Lidan tablets from JK.

Sample No.	Similarity	Sample No.	Similarity
L80	1	L84	0.999
L81	1	L85	0.999
L82	1	L86	1
L83	1		

Table S1. (**G**) The similarity of Xiaoyan Lidan tablets from BH.

Sample No.	Similarity	Sample No.	Similarity
L93	0.999	L96	1
L94	0.999	L97	0.999
L95	0.999		

Table S1. (**H**) The similarity of Xiaoyan Lidan tablets from GF.

Sample No.	Similarity	Sample No.	Similarity
L98	0.992	L100	0.999
L99	1	L101	0.995

Table S1. (**I**) The similarity of Xiaoyan Lidan tablets from JM.

Sample No.	Similarity	Sample No.	Similarity
L102	0.999	L104	0.999
L103	0.999	L105	1

Table S1. (**J**) The similarity of Xiaoyan Lidan tablets from YH.

Sample No.	Similarity	Sample No.	Similarity
L106	1	L108	0.995
L107	1		

Table S1. (**K**) The similarity of Xiaoyan Lidan tablets from QJ.

Sample No.	Similarity	Sample No.	Similarity
L109	0.999	L111	0.999
L110	1		

Table S1. (**L**) The similarity of Xiaoyan Lidan tablets from LS.

Sample No.	Similarity	Sample No.	Similarity
L112	0.999	L113	0.999

Table S2. The Eigenvalues, Percentage and Cum. Percentage of PCA.

	Extraction Sums of Squared Loadings		
Component	Total	% of Variance	Cumulative %
1	14.6	37.5	37.5
2	5.5	13.9	51.5
3	4.1	10.6	62.1
4	3.9	9.9	72.1
5	2.59	6.5	78.6
6	1.99	4.9	83.6
7	1.49	3.6	87.2

Extraction Method: Principal Component Analysis.

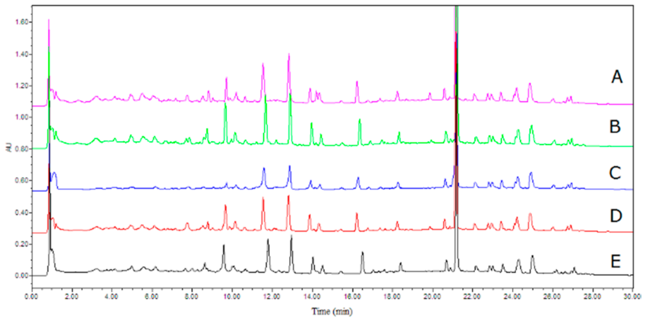

Figure S1. Study of different solvents in extraction: (**A**) methanol; (**B**) hydrochloric acid concentration in methanol (0.5%, *v*/*v*); (**C**) 70% methanol; (**D**) ethanol; and (**E**) 70% ethanol

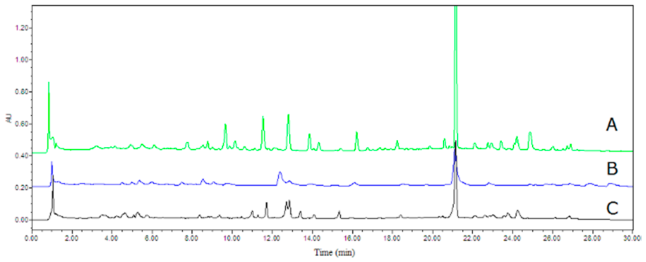

Figure S2. Study of different columns. (**A**) ACQUITY UPLC BEH C_{18} column (100 × 2.1 mm, i.d., 1.7 μm); (**B**) ACQUITY UPLC HSS C_{18} column (100 × 2.1 mm, i.d., 1.7 μm); (**C**) Kinetex 1.7-μm u XB-C_{18} 100A (100 × 2.1 mm, i.d., 1.7 μm).

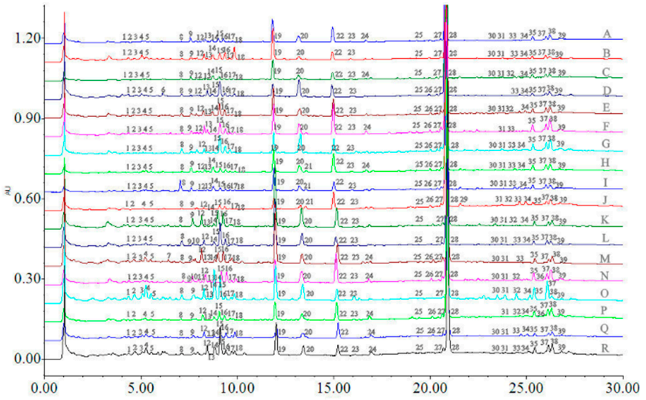

Figure S3. UPLC fingerprint chromatograms of 18 batches of XYLDTs.

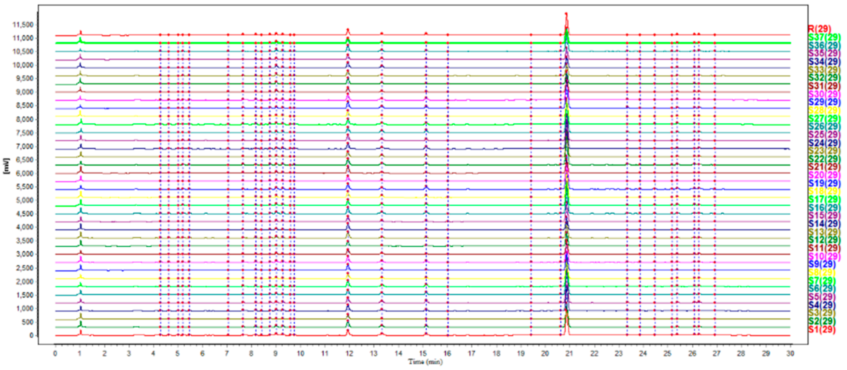

(**A**)

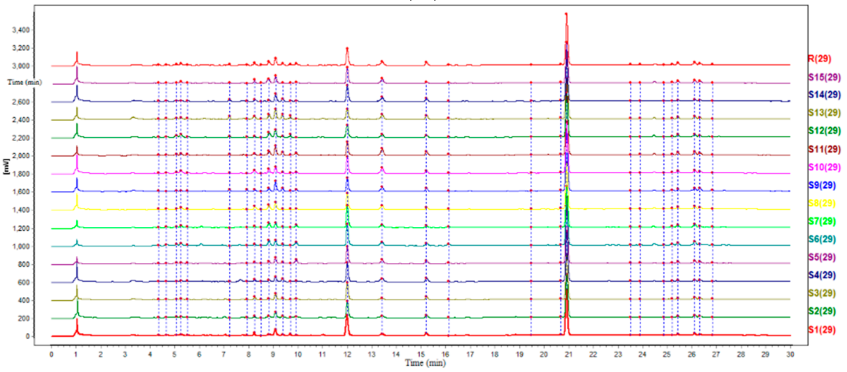

(**B**)

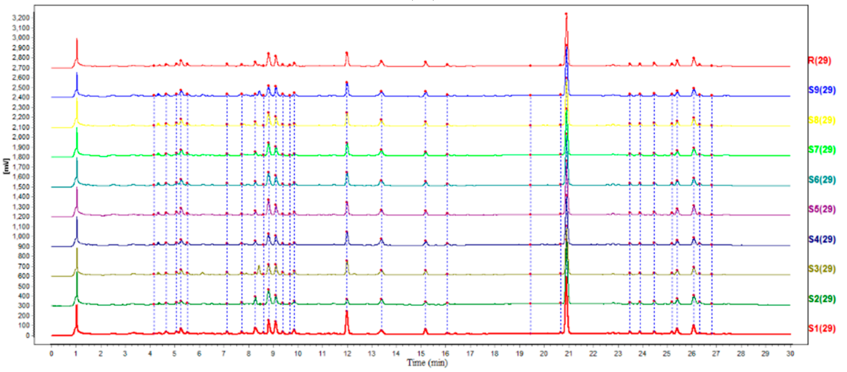

(**C**)

Figure S4. *Cont.*

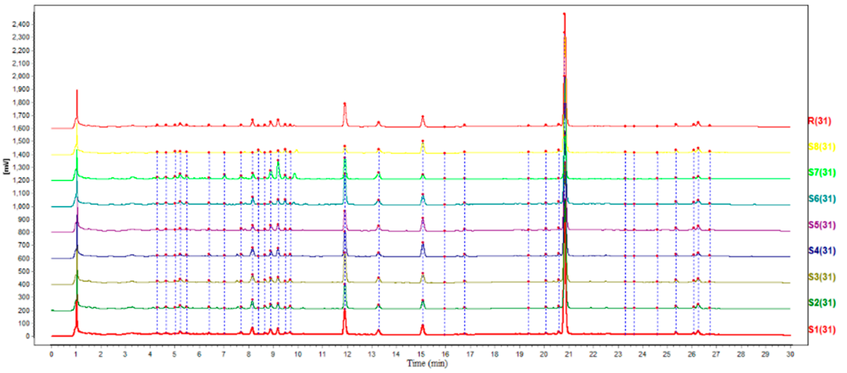

(**D**)

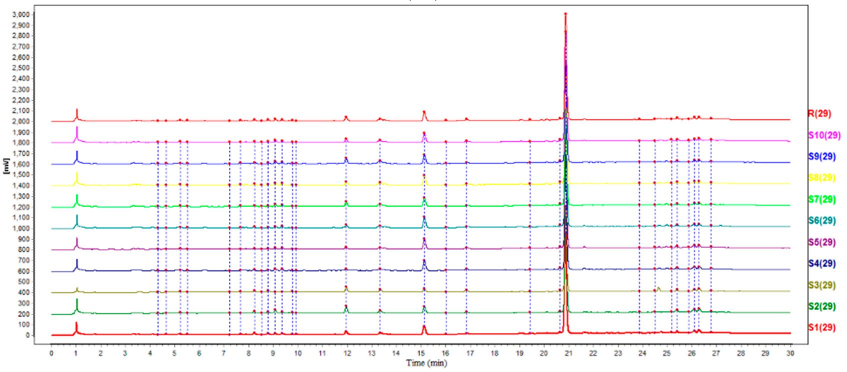

(**E**)

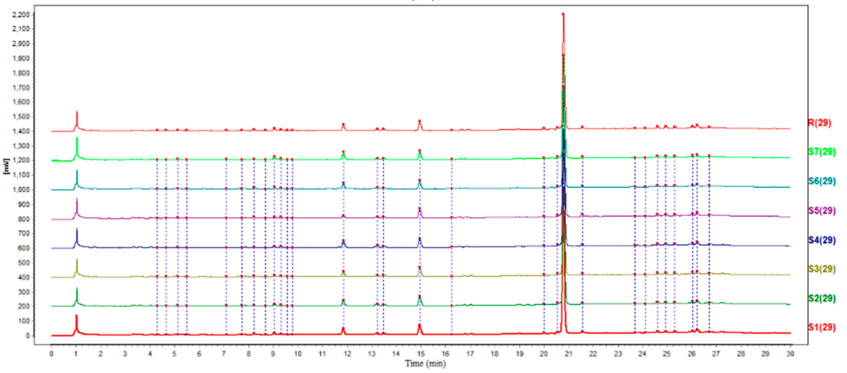

(**F**)

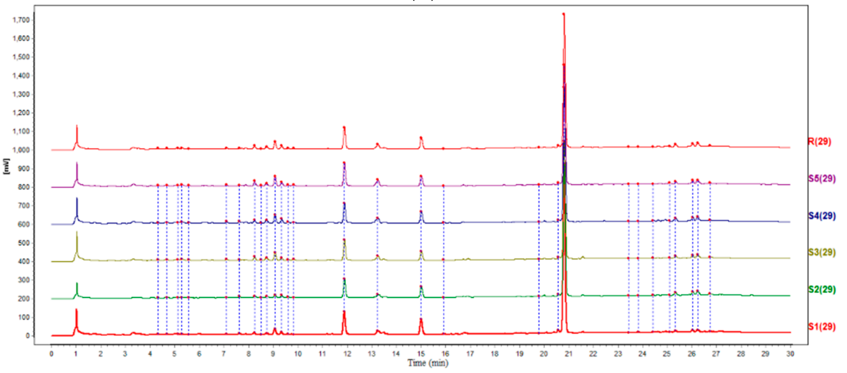

(**G**)

Figure S4. *Cont.*

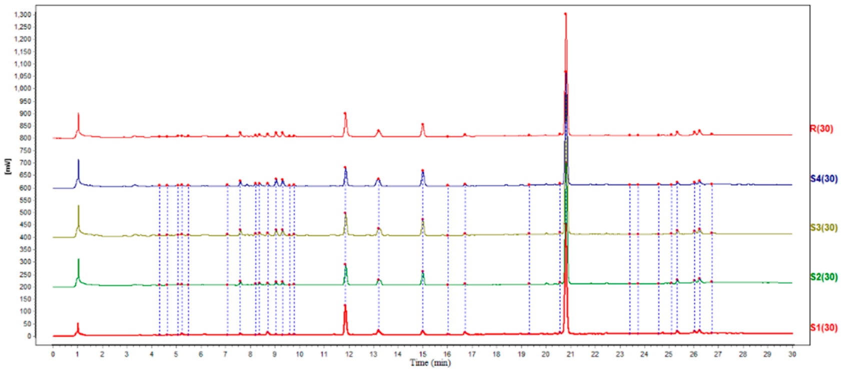

(**H**)

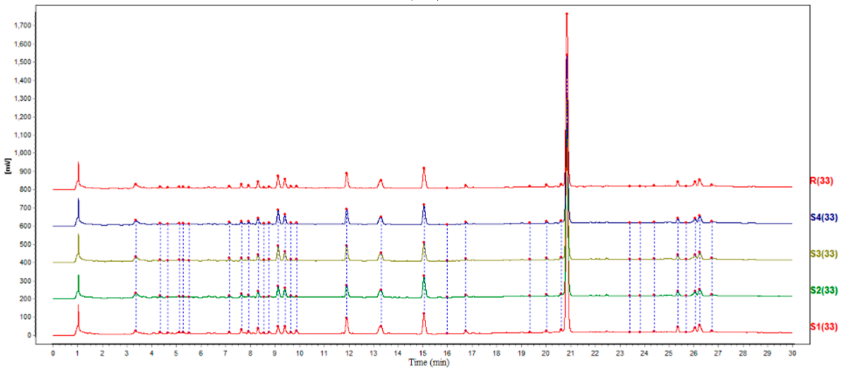

(**I**)

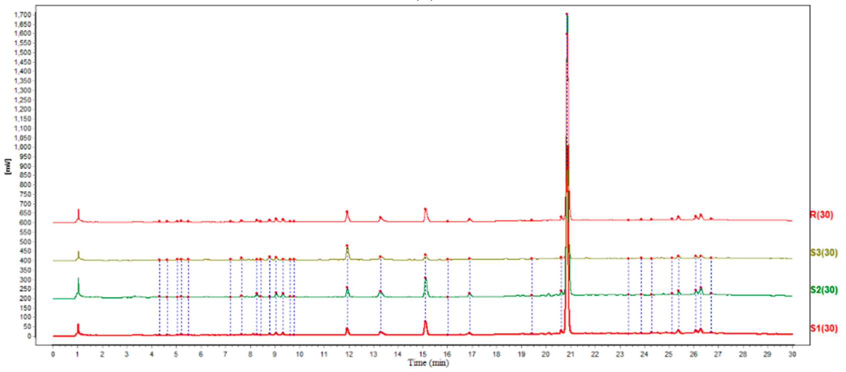

(**J**)

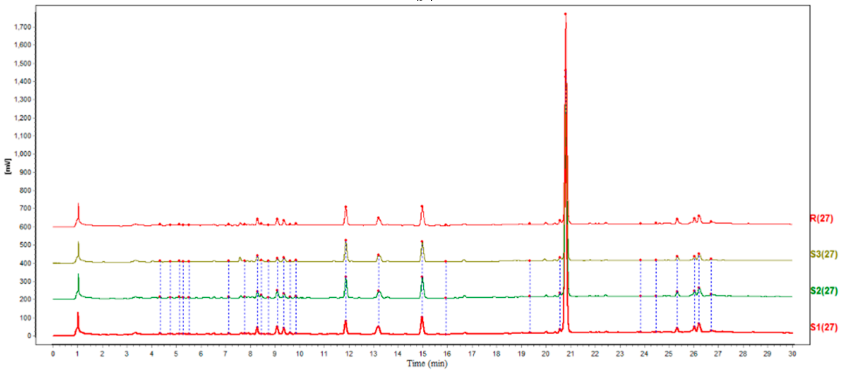

(**K**)

Figure S4. *Cont.*

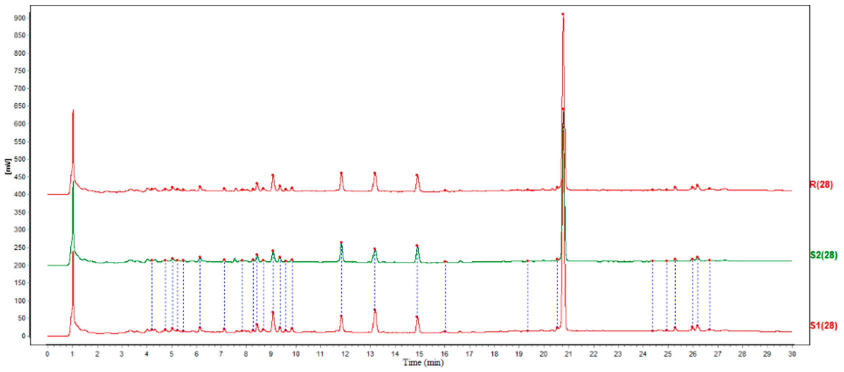

(**L**)

Figure S4. UPLC fingerprint chromatograms of XYLDTs from different manufactures: LFS (**A**); BYS (**B**); WNQ (**C**); JY (**D**); XF (**E**); JK (**F**); BH (**G**); GF (**H**); JM (**I**); YH (**J**); QZ (**K**); and LS (**L**). R: digital standard fingerprint.

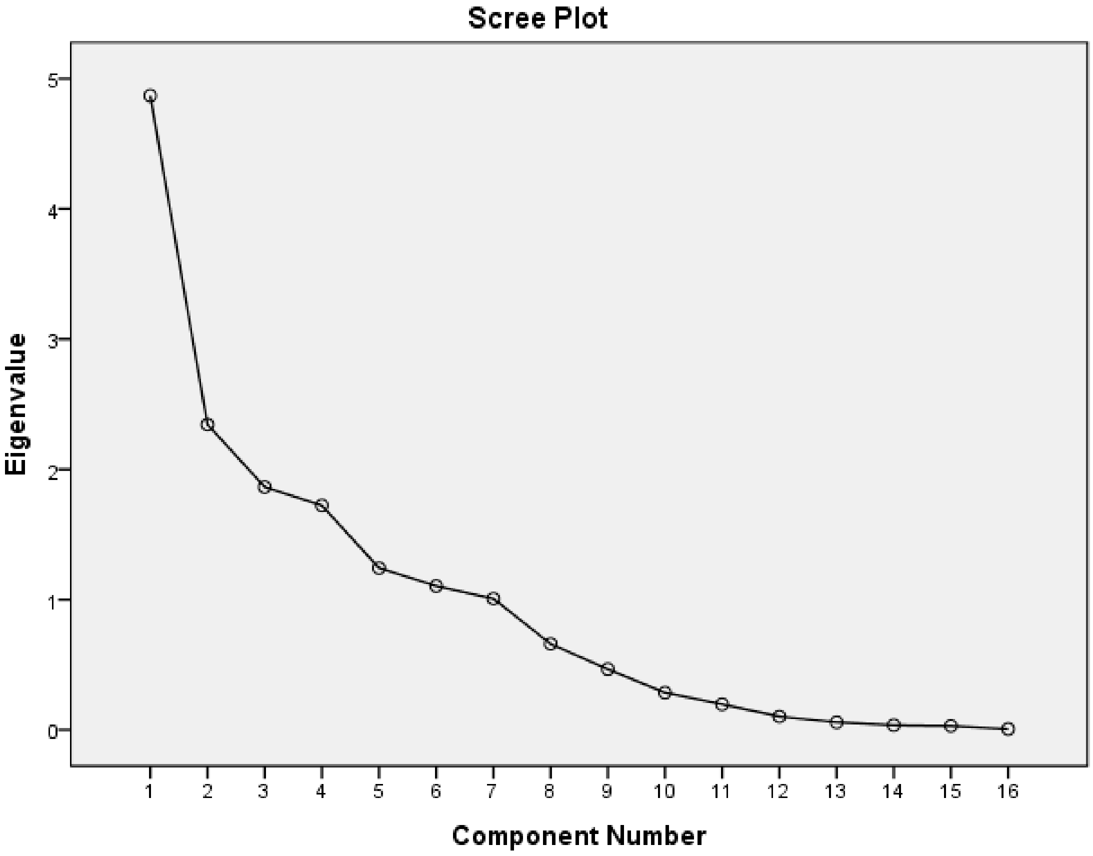

Figure S5. Eigenvalues for the 16 PCs. The first five PCs (PCs 1–5) captured substantial chemical variations.
